# Preparation of Hydrophobic Glass Surfaces by Femtosecond Laser

**DOI:** 10.3390/mi16090988

**Published:** 2025-08-28

**Authors:** Xuyun Peng, Xiaojun Tan, Wei Tan, Jian Huang, Chaojun Ding, Yushan Yang, Jieshun Yang, Haitao Chen, Liang Guo, Qingmao Zhang

**Affiliations:** 1 Sino-German Intelligent Manufacturing School, Shenzhen City Polytechnic, Shenzhen 518116, China; 19970975568@163.com (X.P.); 18620373551@163.com (W.T.); hejson@163.com (J.H.); 18617076210@163.com (C.D.); real210@163.com (Y.Y.); yangjieshun@126.com (J.Y.); a202415814644653@163.com (H.C.); 2 Guangdong Provincial Key Laboratory of Nanophotonic Functional Materials and Devices, School of Information and Optoelectronic Science and Engineering, South China Normal University, Guangzhou 510006, China; guoliangchn@163.com (L.G.); zhangqm@scnu.edu.cn (Q.Z.)

**Keywords:** femtosecond laser, hydrophobic surface, Panda glass, laser parameters, hierarchical micro/nanostructure, contact angle

## Abstract

Functional glass surfaces with tunable wettability are of growing interest in optical, biomedical, and architectural applications. In this study, we investigate the influence of femtosecond laser processing parameters—including power, scanning speed, and repetition rate—on the surface morphology, wettability, and optical properties of Panda glass. Laser structuring generated microscale ablation features and increased surface roughness (arithmetic mean height, Sa, rising from ~0.02 µm for pristine glass to ~1.85 µm under optimized conditions). The treated surfaces exhibited enhanced hydrophobicity, with static water contact angles up to ~82° and sliding angles exceeding 50°, indicating significant droplet pinning. Optical characterization further showed a reduction in transmittance at 550 nm from ~92% (pristine) to ~68% after laser treatment, consistent with increased scattering by surface textures. These findings demonstrate that femtosecond laser processing is an effective mask-free method to enhance the hydrophobicity of glass surfaces and establish clear process–structure–property relationships, providing guidance for future optimization toward superhydrophobic performance.

## 1. Introduction

The demand for functional glass surfaces has grown significantly in recent years, driven by rapid advancements in consumer electronics [1], optical instruments [2], biomedical devices [3], and architectural applications [4]. Among the various types of advanced glass materials, Panda glass stands out due to its exceptional mechanical durability; high optical transmittance; chemical resistance; and thermal stability [5]. These properties make it an attractive candidate for display panels; camera lenses; and precision optics, where surface functionality such as water repellency; anti-fogging; and self-cleaning is increasingly important [6].

Traditional surface treatment methods—such as chemical etching, sandblasting, or sol–gel coating—have demonstrated some success in altering the wettability of glass surfaces [7]. However, these techniques often suffer from limitations such as environmental hazards, lack of durability, poor spatial resolution, and difficulty in controlling micro/nanostructure formation [8,9]. Consequently, there is a growing interest in cleaner, more precise, and scalable alternatives for fabricating functional glass surfaces [10].

Femtosecond laser micromachining offers a non-contact, highly controlled, and localized approach for inducing surface modifications at the micro- and nanoscale [11]. Owing to its ultra-short pulse duration (on the order of 10^−15^ s), femtosecond laser irradiation minimizes thermal diffusion and collateral damage, allowing for the creation of well-defined periodic and hierarchical structures [12]. Such features are known to significantly influence surface wettability. Recent studies have demonstrated remarkable advances in this area. For instance, LIPSS-based texturing on titanium alloys produced superhydrophobic surfaces with water contact angles exceeding 161.6°, along with enhanced anti-corrosion and anti-icing properties [13]. On quartz glass, laser backside ablation combined with PTFE coating yielded a hydrophobic angle of 153 ± 2° using a pulse energy of 12 μJ and scanning speed of 100 mm/s [14]. Similarly, the femtosecond laser etching of glass fiber-reinforced plastics generated periodic micro–nanohole arrays that reached a contact angle of 161°, demonstrating the critical role of ablation pit volume in tuning wetting behavior [15]. A recent study on the laser texturing of glass achieved a balance between high transparency (above 80% transmittance) and hydrophobicity, indicating potential for engineering multifunctional optical surfaces [16]. Together, these advances provide valuable strategies for fabricating periodic and hierarchical structures, which offer feasible pathways for achieving superhydrophobicity and serve as important references for the further optimization of our approach on Panda glass.

Very little is known about how femtosecond laser processing affects the surface structure and wetting behavior of Panda glass, which differs compositionally and structurally from more commonly studied silica- or borosilicate-based glasses. Panda glass is a high-strength optical glass characterized by a composite structure with enhanced mechanical durability, high transparency, and excellent thermal stability, making it attractive for demanding optical and structural applications [17]. These unique properties also suggest that its response to ultrafast laser structuring may deviate significantly from that of conventional glass, warranting dedicated investigation.

Building on these recent advances, this study focuses on Panda glass, a substrate distinguished by its high mechanical strength, optical clarity, and potential for advanced functional applications yet scarcely explored for femtosecond laser-induced wettability control. Unlike prior works that achieved superhydrophobicity through coatings, composite materials, or specialized structuring, our investigation systematically examines how femtosecond laser parameters—including power, scanning speed, and repetition rate—affect the surface morphology, roughness, optical properties, and wettability of Panda glass. By establishing quantitative relationships between process parameters, microstructural features, and hydrophobic performance, this work provides new insight into the feasibility of direct laser-induced wettability modification of glass without chemical coatings. Although the current results achieve only hydrophobicity rather than superhydrophobicity, they lay the groundwork for future optimization strategies, including coupling laser structuring with chemical functionalization, to extend the performance of Panda glass toward practical self-cleaning and optical applications.

## 2. Materials and Methods

### 2.1. Sample Preparation and Cleaning Protocol

Commercially available Panda glass substrates (60 wt% SiO_2_ and 40 wt% Al_2_O_3_, 50 mm × 50 mm × 2 mm), supplied by Sichuan Hongke Innovation Technology Co., Ltd. (Mianyang, China), were selected for this study due to their superior transparency, chemical durability, and mechanical stability. Prior to laser treatment, all samples were subjected to a standardized ultrasonic cleaning process. Each specimen was immersed in deionized (DI) water and absolute ethanol for 15 min each to remove particulate contaminants and surface residues. The cleaned samples were subsequently dried in a convection oven at 60 °C for two hours and stored in a desiccator to avoid moisture adsorption prior to laser processing.

### 2.2. Femtosecond Laser Processing

**Figure 1:**
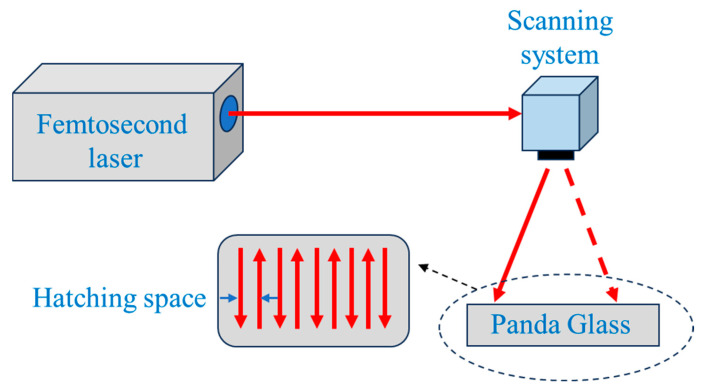
Schematic diagram of processing.

A high-power industrial femtosecond laser system (FemtoYL-40, wavelength: 355 nm, pulse duration: 408 fs, highest power: 30 W) was employed to induce surface structuring. The laser beam was focused onto the sample surface using a galvanometric scanner equipped with a telecentric lens, providing a nominal spot diameter of 2.262 μm (X-axis) and 2.240 μm (Y-axis). The laser scanning trajectory was defined using a custom G-code pattern with a hatch spacing of 45 μm and a defocus distance set to 0 mm, as shown in Figure 1.

To systematically study the influence of key processing parameters, laser power (ranging from 9.60 W to 11.25 W), scanning speed (500–5000 mm/s), and pulse repetition frequency (1000–1450 Hz), as shown in Table 1, were independently varied while maintaining constant hatch spacing and focusing conditions.

### 2.3. Surface Characterization Techniques

Surface morphology and roughness were characterized using white-light interferometry (MarSurf CM mobile, Mahr, Stuttgart, Germany) to capture three-dimensional surface topography. Quantitative roughness parameters including arithmetic mean roughness (Sa) and maximum height (Sz) were extracted from these measurements.

Phase identification and crystallographic structure changes were assessed via X-ray diffraction (XRD) using a Bruker D8 Advance diffractometer equipped with Cu Kα radiation (λ = 1.5406 Å) over a 2θ range of 20° to 120°.

Optical transmittance was measured using a UV–Vis–NIR spectrometer (Thermo Fisher Scientific, Waltham, MA, USA) across a wavelength range of 200–1100 nm to assess changes in transparency post-laser processing.

Morphology observation was carried out on a scanning electron microscope (SEM) (Gemini 300, ZEISS, Peine, Germany), which was equipped with an EDS unit (X-maX, Oxford, UK) to analysis the elemental content.

### 2.4. Wettability Assessment

To evaluate hydrophobic performance, static water contact angles were measured using an optical contact angle goniometer (SL200KS, Boston, MA, USA). For each measurement, an 8 μL droplet of deionized water was dispensed onto the sample surface using a micro-syringe, and the droplet shape was recorded via high-resolution imaging. Contact angle values were averaged over three measurements taken at different points on each sample. Superhydrophobicity is generally defined as a surface state where the static water contact angle exceeds 150°, accompanied by a sliding (rolling) angle smaller than 10°, which together indicate strong water repellency and weak droplet adhesion.

## 3. Results and Discussion

### 3.1. Surface Morphology and Micro/Nanostructure Analysis

#### 3.1.1. Pristine Surface Morphology

**Figure 2:**
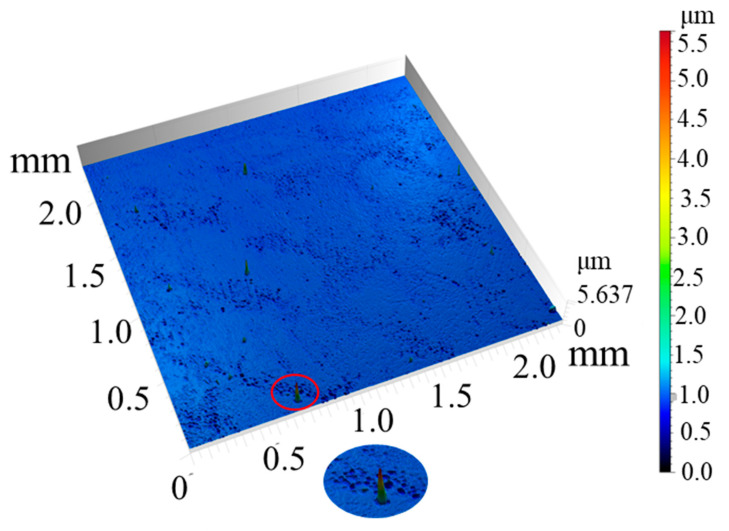
The morphology of untreated Panda glass surface (the enlarged view of protuberance is shown below).

The untreated Panda glass surface exhibited a smooth, nearly featureless topology, as shown in Figure 2. Measured via white-light interferometry, the Sa was approximately 0.0212 μm, while the Sz reached 5.637 μm. The disproportionately high Sz value relative to Sa is attributed to isolated surface asperities, potentially caused by residual particulates or slight manufacturing defects. No micro- or nanoscale features were observed.

#### 3.1.2. Effect of Laser Frequency on Surface Structures

**Figure 3:**
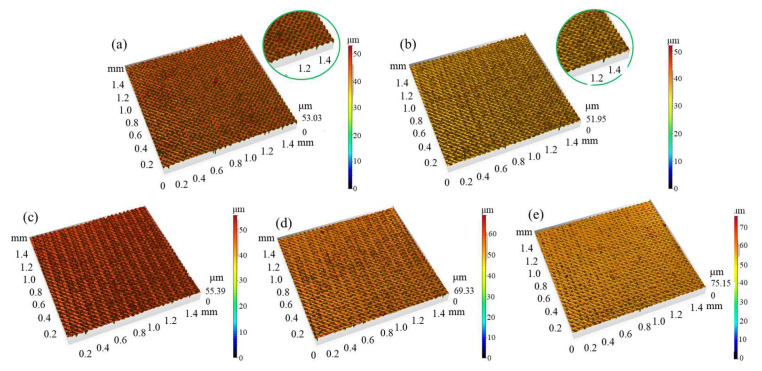
The morphology of Panda glass surface treated by different laser frequencies: (**a**) 1050 Hz; (**b**) 1150 Hz; (**c**) 1250 Hz; (**d**) 1350 Hz; (**e**) 1450 Hz.

**Figure 4:**
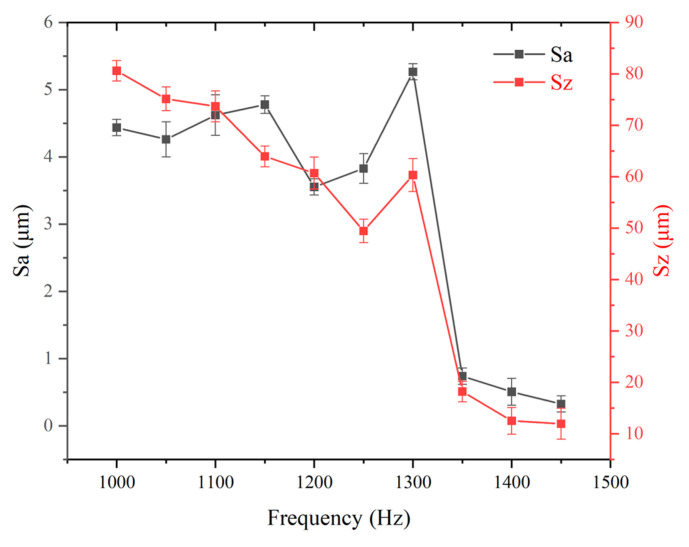
The influence of laser frequency on the Sa and Sz of Panda glass surface.

In this set of experiments, the laser power was fixed at 11.25 W and the scanning speed at 2000 mm/s, while the laser frequency was varied. Figure 3 illustrates the topography of Panda glass surfaces treated under various laser frequencies (1000–1450 Hz), and Figure 4 counts the results. Surface roughness demonstrated a nonlinear relationship with frequency. Sz decreases with frequency, except at 1300 Hz. Sa fluctuates up and down from 1000 Hz to 1300 Hz. After 1300 Hz, it decreases. Both Sa and Sz decrease sharply from 1300 Hz to 1350 Hz.

When the laser frequency increases, the average laser power increases. The average laser power can be calculated by the following equation:

P_ave_ = E × f (1)

where P_ave_ is the average laser power, E is the laser pulse energy, and f is the laser frequency.

There exists a laser power threshold, when P_ave_ is higher than the laser power threshold, where the Panda glass surface is severely vaporized and Sa/Sz are quite low. This laser power threshold may happen between 1300 Hz and 1350 Hz. At lower laser power, the surface exhibits features indicative of partial melting with localized ablation, rather than a uniform vaporization-dominated regime. This suggests that both melting and vaporization mechanisms may coexist in this transitional region. The zoomed-in inset in Figure 3 highlights these mixed features more clearly, showing shallow melt layers adjacent to localized ablation pits. This results in Sa being unstable from 100 Hz and 1300 Hz. Especially for a laser frequency of 1300 Hz, vaporization is greater than melting, which weakens the effect of peak reduction and valley filling. That is why both Sa and Sz form a spike at 1300 Hz.

#### 3.1.3. Effect of Laser Power on Surface Structures

**Figure 5:**
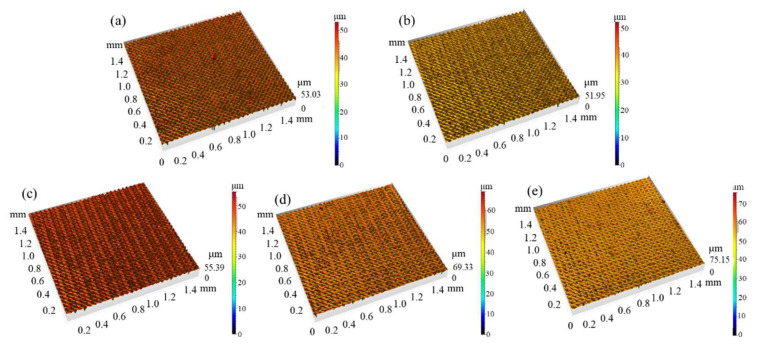
Surface morphology of Panda glass treated with different laser powers: (**a**) 9.60 W, (**b**) 9.75 W, (**c**) 9.90 W, (**d**) 10.05 W, (**e**) 10.20 W. SEM images show the evolution of pit depth, splash formation, and micro/nanotexture sharpness as laser power increases.

**Figure 6:**
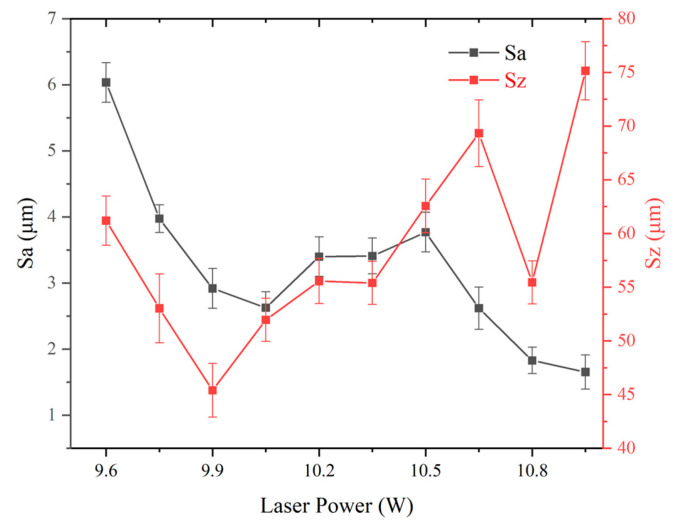
Effect of laser power on surface roughness parameters of Panda glass. The plot shows arithmetic mean roughness (Sa, black curve) and maximum height (Sz, red curve) as functions of laser power. Data points represent the average of three measurements; error bars indicate standard deviation.

Here, the laser frequency was maintained at 1300 kHz and the scanning speed at 2000 mm/s, while the laser power was adjusted. Figure 5 reveals a complex dependency of surface morphology on laser power. Figure 6 counts the results.

The Sa decreases from 9.60 W laser power to 10.05 W laser power and then increases until 35% laser power is reached. It finally decreases from 10.50 W laser power to 10.95 W laser power. The Sz first decreases and then increases, and finally it fluctuates up and down from 10.50 W laser power to 10.95 W laser power.

The glass vaporization laser power threshold occurs at 10.50 W laser power; beyond 10.50 W laser power, the glass surface severely vaporizes and the Sa decreases. Below 9.90 W laser power, the reaction between the laser and the glass surface is melting; the laser power increases and the Sa and Sz decrease. This is due to the effect of peak reduction and valley filling of melting. Between 9.90 W and 10.50 W, both melting and vaporization exist, the effect of peak reduction and valley filling of melting is weakened, and Sa and Sz increase with laser power as this increases from 10.05 W to 10.50 W.

These results highlight the importance of power optimization in achieving functional textures.

#### 3.1.4. Effect of Scanning Speed on Surface Structures

**Figure 7:**
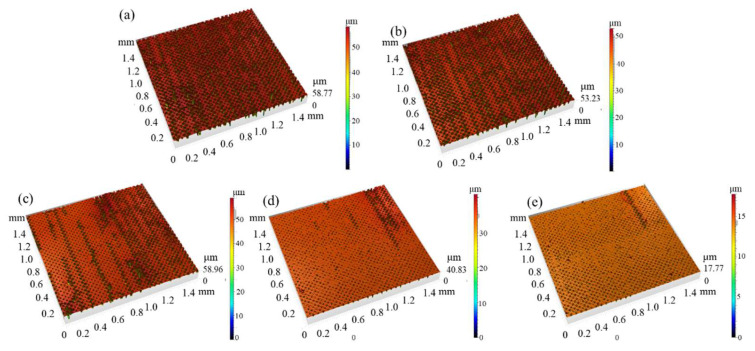
Surface morphology of Panda glass treated at different scanning speeds: (**a**) 1000 mm/s, (**b**) 2000 mm/s, (**c**) 3000 mm/s, (**d**) 4000 mm/s, (**e**) 5000 mm/s. SEM images reveal changes in pit uniformity, depth, and surrounding splash features with increasing speed.

**Figure 8:**
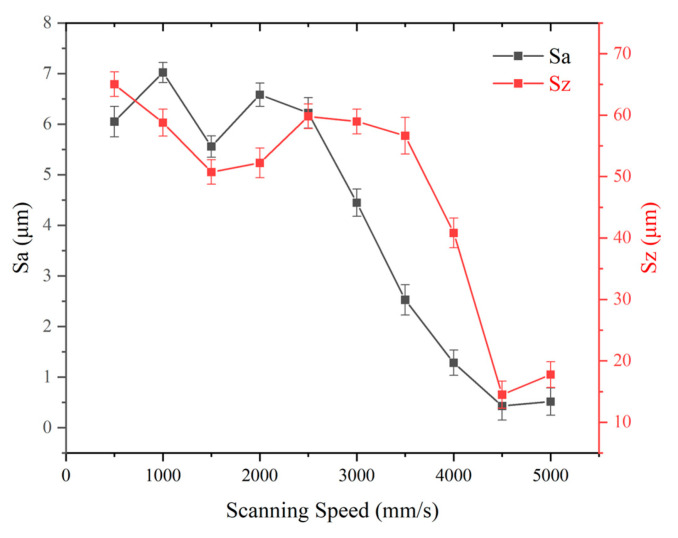
Effect of scanning speed on surface roughness parameters of Panda glass. The plot shows arithmetic mean roughness (Sa, black curve) and maximum height (Sz, red curve) as functions of scanning speed. Data points are the mean of three measurements; error bars represent standard deviation.

For these experiments, the laser power was set at 11.25 W and the repetition frequency at 1300 kHz, while the scanning speed was varied systematically. Figure 7 illustrates surface changes under varying scanning speeds (500–5000 mm/s), and Figure 8 counts the results.

At scanning speeds under 2500 mm/s, Sa and Sz are high and they fluctuate up and down. Beyond 2500 mm/s, Sa and Sz decrease: Sa decreases sharply from 2500 mm/s to 4500 mm/s, and Sz decreases sharply from 3500 mm/s to 4500 mm/s. When the laser scanning speed continues to increase from 4500 mm/s to 5000 mm/s, Sa and Sz increase a little.

At low scanning speeds, the laser works longer at each point, resulting in deeper holes; thus, Sa and Sz are high. Sa and Sz fluctuate up and down, perhaps due to the complexity of the reaction of the laser with the glass surface. Beyond 2500 mm/s, the holes become shallow and Sa/Sz decrease.

### 3.2. Optical Properties and Light Absorption

#### 3.2.1. Laser Frequency and Optical Absorbance

**Figure 9:**
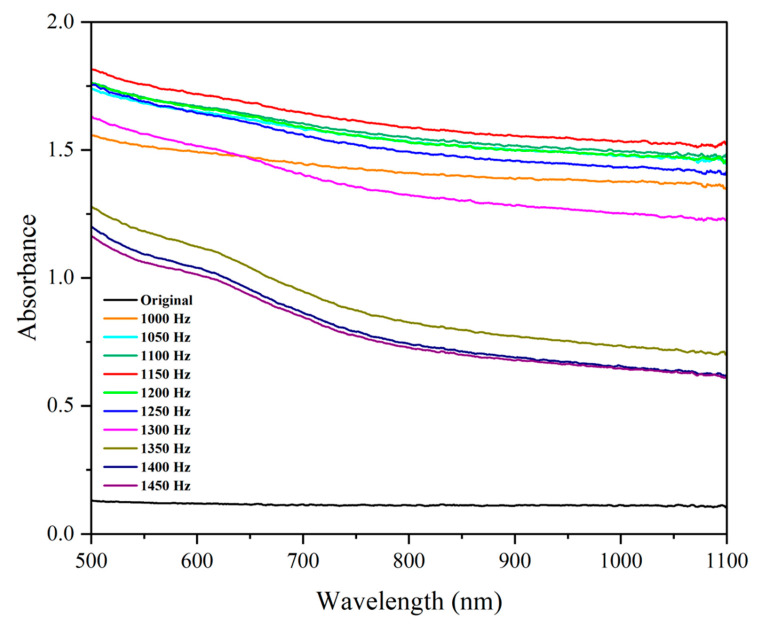
The influence of laser frequency on the absorbance of the surface.

As shown in Figure 9, all laser-treated samples demonstrated higher absorbance than untreated glass in the 500–1100 nm wavelength range. This is due to increased surface roughness and scattering effects. Notably, surfaces processed at 1150 Hz displayed peak absorbance, which can be attributed to subwavelength feature spacing in the range of approximately 400–600 nm, comparable to visible light wavelengths and favorable for light trapping. This observation is consistent with prior reports that subwavelength surface textures enhance light absorption through multiple scattering and diffraction effects [18].

Absorbance trends showed a general decline with increasing wavelength, consistent with the Mie scattering theory [19]. Compared to the pristine sample—which remained nearly transparent across the spectrum—laser-treated specimens exhibited broadband absorption, which is desirable in anti-reflective or solar cell applications.

#### 3.2.2. Effect of Laser Power on Optical Behavior

**Figure 10:**
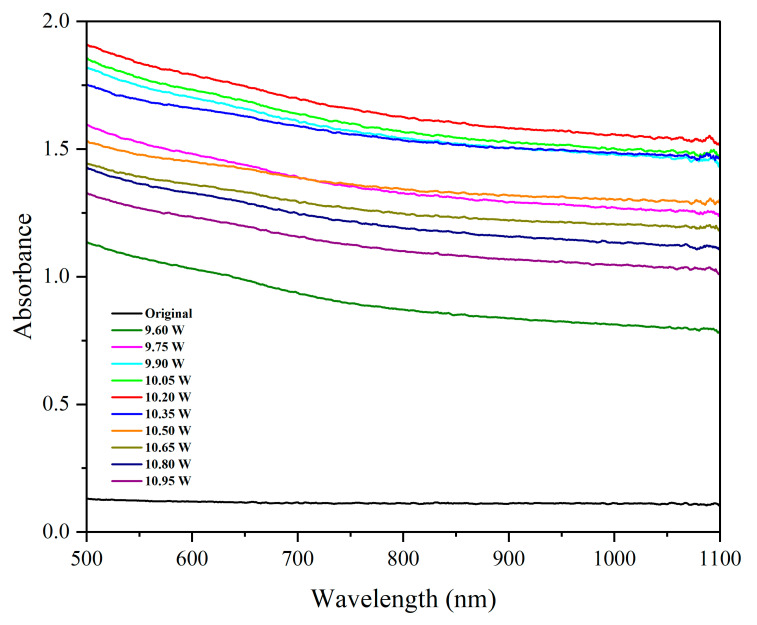
The influence of laser power on the absorbance of the surface.

Figure 10 highlights how absorbance varies with laser power. The absorbance rose sharply as power increased to 10.20 W and then decreased. Surface features formed at 10.20 W laser power appeared to optimize light–matter interaction, inducing enhanced multiple reflections and localized plasmonic effects (for submicron features).

Beyond this point, melting-induced smoothing reduced the density and sharpness of the structures, lowering absorbance. These observations further underscore the need for fine-tuning laser fluence to balance structure formation and thermal side effects.

#### 3.2.3. Effect of Scanning Speed on Absorbance

**Figure 11:**
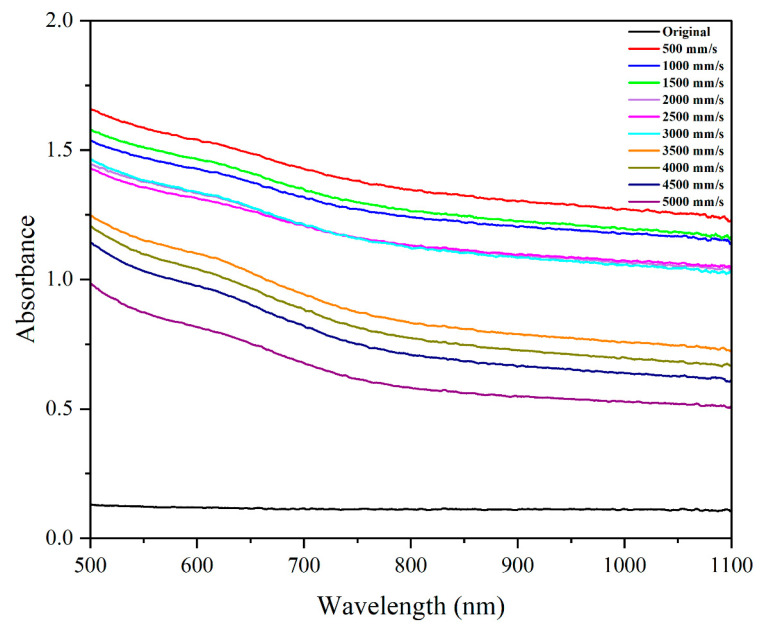
The influence of laser speed on the absorbance of the surface.

As shown in Figure 11, absorbance trends as a function of scanning speed largely mirrored the patterns seen in roughness metrics. Absorbance generally decreased with higher scanning speeds, reflecting the lower surface roughness and diminished nanofeature presence. Interestingly, a local maximum was observed at 500 mm/s in the visible spectrum (500–750 nm), corresponding to conditions where microstructure uniformity and depth were optimized.

The dependence of optical properties on topography suggests that femtosecond laser structuring can serve as a versatile tool not only for wettability control but also for tailoring light–matter interactions in photonic devices.

### 3.3. Wettability and Hydrophobicity Performance

#### 3.3.1. Pristine Surface Wettability

**Figure 12:**
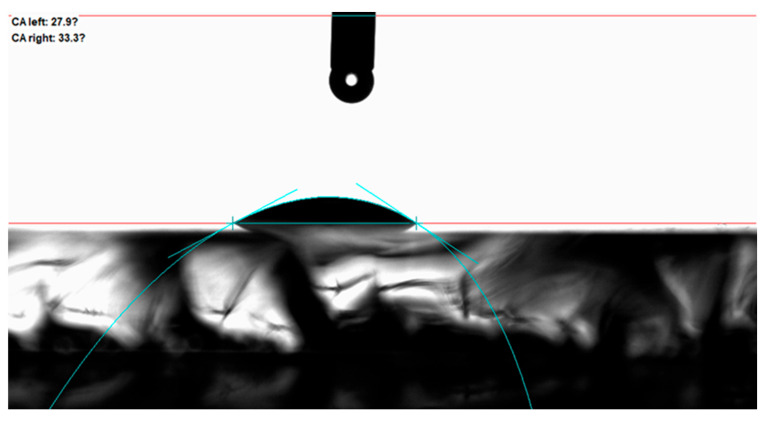
The contact angle of the original surface.

Figure 12 shows that the untreated Panda glass had a contact angle of approximately 30°, categorizing it as highly hydrophilic. This is typical for smooth glass surfaces, which promote complete wetting due to strong surface energy and the absence of air-trapping geometries [20].

#### 3.3.2. Influence of Laser Frequency on Wettability

**Figure 13:**
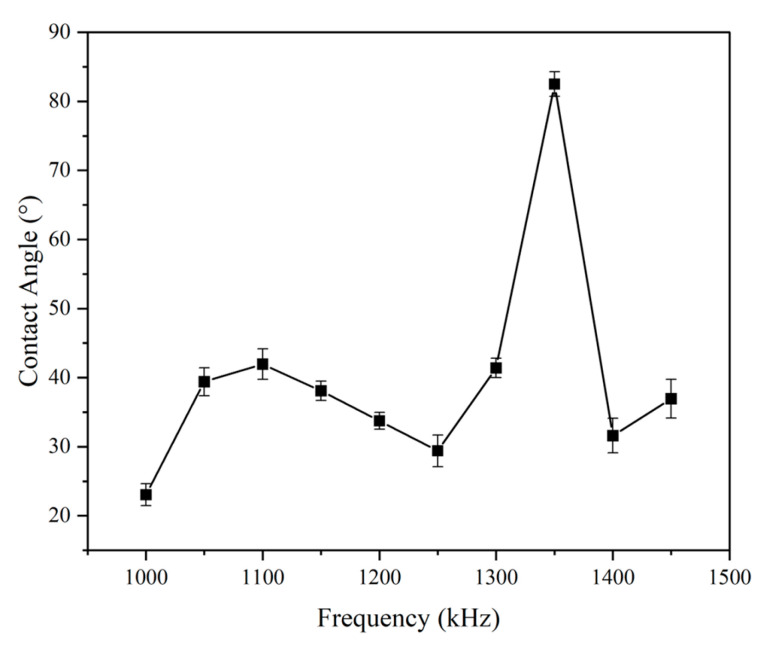
Contact angle results as a function of laser frequency (laser power = 11.25 W, scanning speed = 2000 mm/s). Error bars represent standard deviation from three independent measurements.

**Figure 14:**
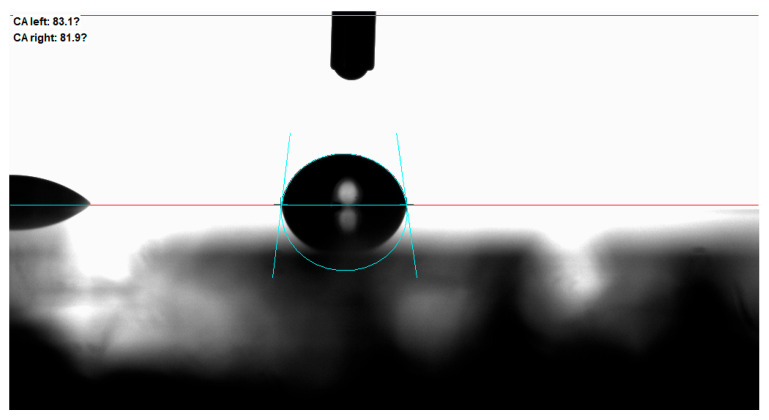
The contact angle of the surface with a laser frequency of 1350 Hz.

Laser frequency had a pronounced effect on water repellency, as illustrated in Figure 13 and Figure 14. The contact angle peaked at ~82° for surfaces processed at 1350 Hz. Although this is significantly higher than the untreated sample, it still falls short of superhydrophobic criteria (>150°). This suggests that while hierarchical textures were forming, they may have lacked the sharpness or spacing required for full Cassie–Baxter wetting [21].

Further chemical modification or reprocessing could be explored to enhance performance. Nonetheless, the trends affirm that intermediate frequencies strike a balance between feature formation and thermal degradation.

#### 3.3.3. Influence of Laser Power on Wettability

**Figure 15:**
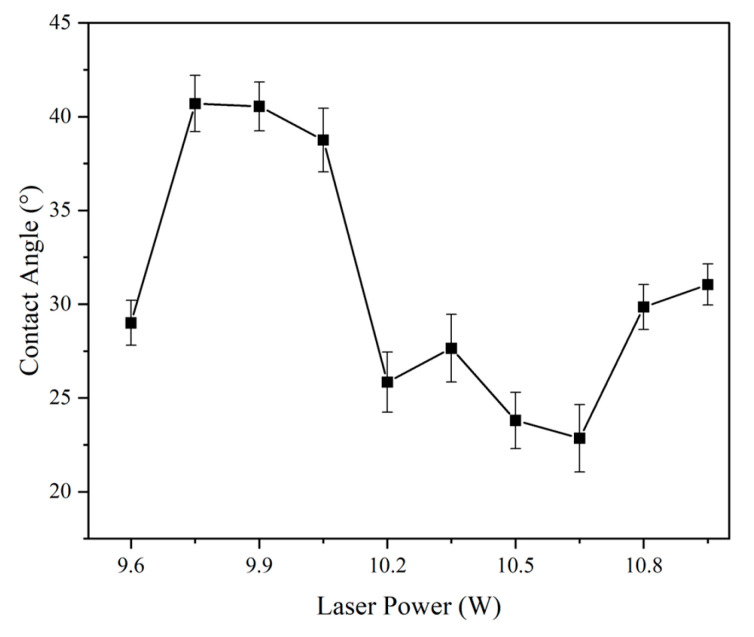
Contact angle results as a function of laser power (laser frequency = 1300 kHz, scanning speed = 2000 mm/s). Error bars represent standard deviation from three independent measurements.

Figure 15 shows that the highest contact angle (~41°) was achieved at 32.5% power, followed by a sharp drop beyond 33.5%, where excessive melting occurred. These findings reinforce earlier observations: excessive thermal input can collapse structured features, diminishing the ability to trap air and repel water droplets.

The correlation between surface morphology and wettability validates the Wenzel and Cassie–Baxter models, suggesting that hydrophobicity is closely linked to optimized roughness at multiple scales [22].

#### 3.3.4. Influence of Scanning Speed on Wettability

**Figure 16:**
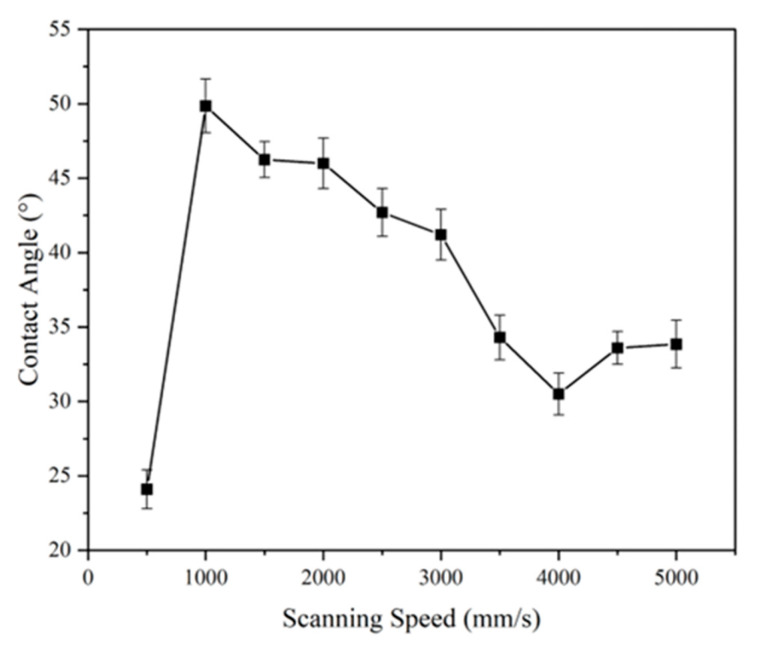
Contact angle results as a function of scanning speed (laser power = 11.25 W; laser frequency = 1300 kHz). Error bars represent standard deviation from three independent measurements.

As shown in Figure 16, contact angles peaked at ~50° for samples processed at 1000 mm/s. Similarly to laser power and frequency, scanning speed also followed a nonlinear influence: low speeds caused thermal effects, while overly high speeds failed to ablate significant structures.

Interestingly, at 500 mm/s, the contact angle dropped below that of the untreated sample, likely due to surface damage or debris deposition, creating a more wettable topology.

### 3.4. XRD, Microstructure, and Sliding Angle

The pristine surface and laser-treated surface by 1350 Hz and 11.25 W were analyzed by XRD, SEM, and EDS, and the sliding angle was also tested.

#### 3.4.1. XRD

**Figure 17:**
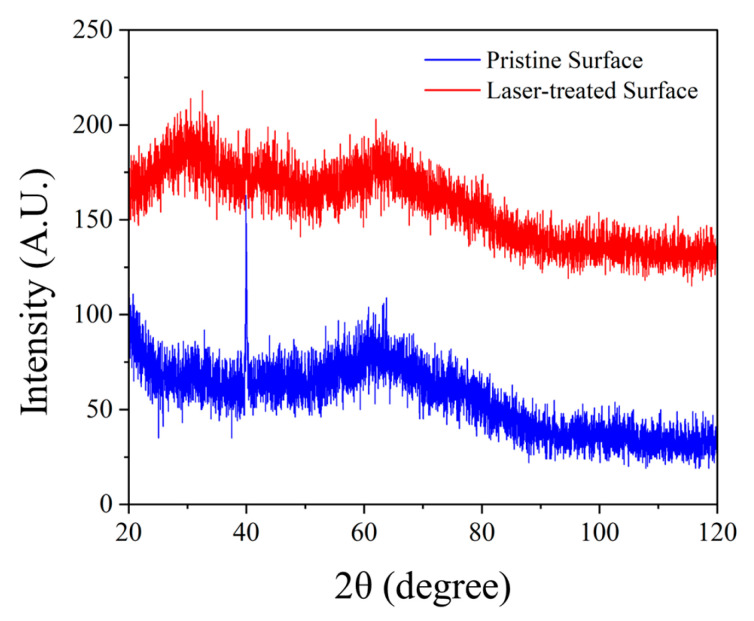
The XRD results of pristine surface and laser-treated surface.

The high intensity of diffraction peaks on the pristine surface indicates that the material has a high crystallinity or ordered structure, as shown in Figure 17. The main diffraction peaks may correspond to the crystal phases of SiO_2_ and Al_2_O_3_, which is consistent with the chemical composition of Panda glass.

The diffraction peak intensity of the laser-treated surface is significantly reduced, indicating that laser processing may lead to partial amorphization of the material or the failure of the lattice structure. Some diffraction peaks may disappear or be displaced, suggesting that laser treatment may have induced phase transitions or formed new metastable structures.

Laser treatment significantly alters the crystal structure of Panda glass, potentially leading to amorphous or microcrystalline formation through high-temperature melting and rapid cooling processes. This structural change can affect the material’s mechanical properties, optical properties, or chemical stability.

#### 3.4.2. Microstructure

**Figure 18:**
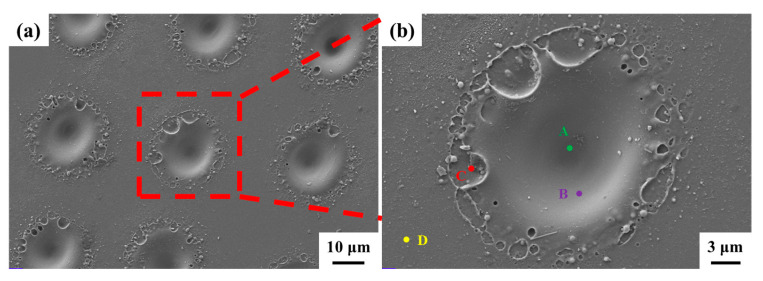
The SEM image of the laser-treated surface (**a**); (**b**) enlarged view of the pit.

The SEM results are shown in Figure 18; the laser-processed pits are distributed in an array. Figure 18b shows the magnified image of one pit. The pit is a smooth cone which is consistent with the energy distribution of the Gaussian beam, and the pit is surrounded by splashed or solidified glass liquid. This different morphology of the pit and its surrounding area can be explained by the fact that the main chemical reaction of the pit center is vaporized and its surrounding area has some melting area.

EDS point scan results of each area of the pit are shown in Table 2. A is the central position of the cone, B is the position of the inner wall of the cone, C is the edge position of the cone, and D is the pristine area. Compared to D (the pristine area), A is rich in Al and O; because SiO_2_ has a lower melting and boiling point than Al_2_O_3_, the evaporation of SiO_2_ results in poor Si. B is rich in oxygen and poor in Si, indicating that oxidation has occurred. C and D show almost the same chemical composition.

#### 3.4.3. Sliding Angle

**Figure 19:**
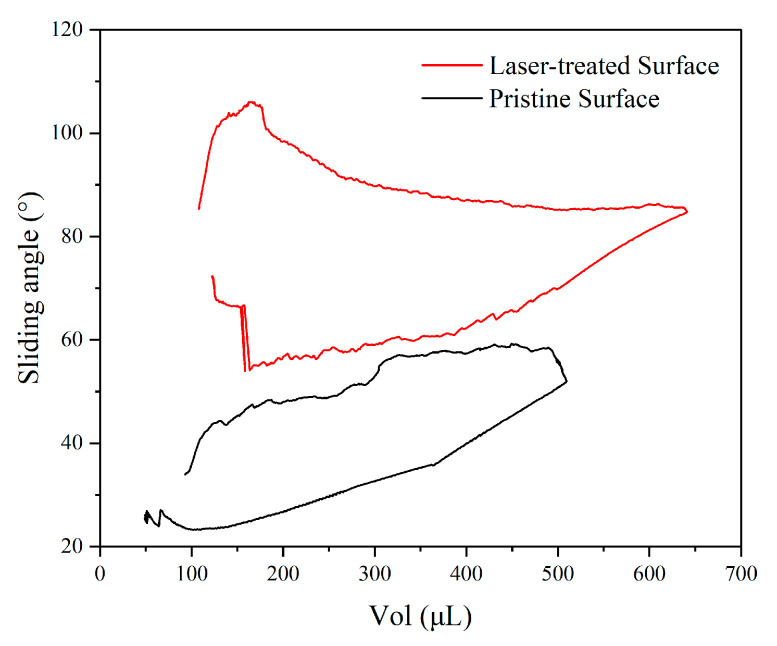
The sliding angles of the pristine surface and laser-treated surface.

Figure 19 shows the sliding angles of the pristine surface and laser-treated surface; the sliding angles of the laser-treated surface are significantly higher than the pristine surface. The videos of the dynamic contact angle can be seen in the Supplementary Materials. The sliding angles of the laser-treated surface are more than 50°; the highest value reaches almost 110°. The sliding angles of the pristine surface are lower than 60°; the lowest value is almost 20°. Laser processing forms micron-scale bulges or closed pores, triggering the Cassie–Baxter state (air cushion effect), resulting in hydrophobicity. If the surface roughness is low/convex, this may enhance the contact angle’s hysteresis, which is manifested as “apparent hydrophobicity”.

## 4. Conclusions and Future Work

### 4.1. Conclusions

This study systematically investigated the preparation of hydrophobic surfaces on Panda glass substrates using femtosecond laser structuring. The effects of three key laser processing parameters—frequency, power, and scanning speed—on the resulting surface morphology, optical properties, and wettability were thoroughly explored.

The key conclusions are as follows:

1. Surface Morphology Evolution:

Femtosecond laser irradiation enabled the formation of hierarchical micro/nanostructures on the glass surface. The morphology varied sensitively with changes in laser frequency, power, and scanning speed. Optimal parameters (e.g., frequency of ~1350 Hz, power around 34%, and speed of ~3000 mm/s) yielded pronounced surface textures that are essential for enhancing surface functionalities.

2. Optical Absorbance Characteristics:

Structured surfaces demonstrated significantly higher light absorbance across the UV–Vis–NIR spectrum compared to pristine Panda glass. This enhancement is attributed to increased scattering and light-trapping effects resulting from micro/nanotexturing. Surfaces processed under intermediate laser fluence exhibited the highest absorbance, indicating optimal structural sharpness and periodicity.

3. Wettability Control:

While complete superhydrophobicity (>150° contact angle) was not achieved, the laser-processed samples exhibited substantial improvements in hydrophobicity, reaching contact angles up to ~82°. The contact angle trends were consistent with the Cassie–Baxter wetting regime and strongly correlated with roughness parameters, confirming that laser-induced hierarchical structures play a central role in altering wetting behavior.

4. Process–Structure–Property Relationship:

This study established clear and quantitative links between processing parameters, surface microstructure, and functional properties (optical and wetting). The results emphasize that the careful tuning of laser parameters is critical for achieving target surface functionalities, whether for light absorption, anti-reflection, or water repellency.

In sum, this work demonstrates that femtosecond laser technology is a promising method for tailoring the surface properties of hard, brittle materials such as Panda glass. The ability to fabricate micro/nanostructured surfaces in a single step, without masks or chemical etching, offers advantages for potential industrial applications. However, given that the maximum contact angle achieved (~82°) remains in the hydrophobic range, further optimization and possible post-treatment are required to achieve superhydrophobicity.

### 4.2. Future Work

Although the present study offers promising insights, several avenues remain open for further exploration.

1. Achieving Superhydrophobicity (>150° Contact Angle):

While our current work achieved only hydrophobicity, future optimization could focus on coupling femtosecond laser-induced micro/nanostructuring with surface energy modification to move toward true superhydrophobicity. This is a technically challenging task, as the modification method must not only reduce surface energy but also preserve optical transparency and structural durability. Recent studies have provided promising approaches in this direction. For example, the ultrafast laser direct-writing of dispersed micro-pit arrays followed by fluorosilane modification with 1H,1H,2H,2H-perfluorodecyltrimethoxysilane (FOTS) achieved transparent superhydrophobic glass surfaces with a water contact angle of 161° and excellent thermostability [22]. Similarly, grid-like micro/nanostructures fabricated by femtosecond laser and subsequently modified with FOTS reached a contact angle of 151° while also exhibiting photothermal de-icing and self-cleaning properties [23]. These strategies provide feasible references for future work, and our ongoing efforts will explore the integration of laser structuring with fluorosilane modification to extend the hydrophobic performance of Panda glass toward practical superhydrophobic applications.

2. Durability and Environmental Stability:

For practical applications, long-term performance under environmental exposure (e.g., UV radiation, abrasion, and humidity) is crucial. Systematic testing of the durability of laser-induced structures and their hydrophobicity over time is needed to evaluate commercial feasibility.

3. Mechanism Study via Modeling and Simulation:

While empirical trends have been observed, detailed thermal–mechanical modeling (e.g., two-temperature models, finite element analysis) could further elucidate the dynamics of laser–glass interaction and help predict optimal processing windows with higher accuracy.

4. Multifunctionality and Cross-application Integration:

Future research could explore combining superhydrophobicity with additional functionalities such as anti-reflection, anti-fogging, or self-cleaning properties. Such multifunctional surfaces could find applications in solar panels, optical sensors, and biomedical devices.

5. Alternative Substrates and Broader Material Compatibility:

Though Panda glass was the focus, extending the approach to other types of glasses (e.g., borosilicate, fused silica) or transparent polymers could significantly broaden the scope of applications and help identify substrate-specific structuring strategies.

6. Scalability and Process Automation:

Investigations into high-throughput patterning methods—such as galvanometer scanning or beam shaping—will be necessary to scale up the process for industrial production. Additionally, integrating in-line monitoring techniques could allow for real-time feedback and process optimization.

Beyond improving wettability performance, the development of transparent superhydrophobic Panda glass also opens up opportunities for multifunctional optical applications. For instance, the femtosecond laser fabrication of microlens arrays on glass has been shown to enable clear imaging with tunable focal length [24]. Coupling such optical functionalities with superhydrophobic surfaces could yield advanced devices that combine imaging or light management with self-cleaning and antifouling properties. Potential application scenarios include outdoor optical sensors, protective windows for imaging systems, and optical components in harsh environments where both clarity and durability are critical.

In conclusion, the femtosecond laser micro/nanostructuring technique represents a versatile platform for next-generation functional surface engineering. With continued optimization, it has the potential to transform how we design optical, wetting, and tribological properties on glass and related materials for diverse technological applications.

## Figures and Tables

**Table 1:** Experimental parameters used for femtosecond laser structuring.

Laser Power (W)	9.60	9.75	9.90	10.05	10.20	10.35	10.50	10.65	10.80	10.95
Scanning speed (mm/s)	500	1000	1500	2000	2500	3000	3500	4000	4500	5000
Frequency (Hz)	1000	1050	1100	1150	1200	1250	1300	1350	1400	1450

**Table 2:** EDS point scan results of each area of the pit.

Element	O	Al	Si
A	69.37	9.47	21.16
B	71.67	6.95	21.38
C	65.73	6.83	27.44
D	65.84	6.79	27.37

## Data Availability

The original contributions presented in this study are included in the article. Further inquiries can be directed to the corresponding author.

## Supplementary Materials

The following supporting information can be downloaded at: https://www.mdpi.com/article/10.3390/mi16090988/s1.
